# Dynamic response of a coal rock caving impact tail beam for hydraulic support

**DOI:** 10.1038/s41598-022-15845-9

**Published:** 2022-07-07

**Authors:** Lijuan Zhao, Liguo Han, Haining Zhang, Xin Jin, Tiangu Wu, Shijie Yang

**Affiliations:** 1 School of Mechanical Engineering, Liaoning Technical University, Fuxin, 123000 China; 2 The State Key Lab of Mining Machinery Engineering of Coal Industry, Liaoning Technical University, Fuxin, 123000 China; 3 Liaoning Province Large Scale Industrial and Mining Equipment Key Laboratory, Fuxin, 123000 China

**Keywords:** Mechanical engineering, Mineralogy

## Abstract

Based on the two-way coupling technology of Discrete Element Method-Multi Flexible Body Dynamics (EDM-FMBD), a virtual caving coal wall is established by using the discrete element software, EDEM. The rigid flexible coupling model of the tail beam of caving supports is established by using multibody dynamics software, RecurDyn. The stiffness of the oil cylinder is calculated by using the solid–liquid spring coupling theory and is replaced by a spring. By simulating the process of a coal rock collapse impacting the tail beam, the dynamic signal from the coal rock collapse impacting the tail beam to crushing in the coal caving stage of the comprehensive caving working face is studied, and the test is carried out underground. The angular acceleration at the hinge point of the tail beam is the largest and shows a variation pattern of "large at both ends and small in the middle". The definition of a "low amplitude band" on the surface of the tail beam is proposed. The force signal at the hinge point of the front link is the strongest and is the best measurement point for the force sensor; the angular acceleration signal at the hinge point of the tail beam is the strongest and it is the best measurement point for the angular acceleration sensor. The results have practical implications for the identification of the coal gangue and the adaptive control of support for integrated top coal mining.

## Introduction

Intelligent mining has become the development direction and the trend of safe and efficient coal mining ^1–3^. The coal caving automation process in comprehensive release mining can not only improve the extraction rate of top coal in the comprehensive release working face and reduce the gangue rate but it can also protect the safety of coal caving workers^4–6^, and the dynamic response of collapsed coal rock for hydraulic support is the key to the accurate identification of fully mechanized caving coal rock. Scholars at home and abroad have conducted extensive research on fully mechanized coal technology and equipment. Jonathan et al.^7^ summarized the development status of underground automation technology and proposed that accurate positioning of mining equipment and the detection of coal seam geological structures are the keys to underground automatic mining. Pytlik et al.^8^ The dynamic response of the hydraulic support column and the test results can be used to determine the yield limit of the column and the optimal design of the safety valve. Hargrave et al.^9^ studied the positioning system of a coal mining machine and tested it underground. Xu Yajun et al.^10^ defined and divided the mechanical equilibrium zone and the bearing capacity of the support according to the different situations of supporting the roof, and studied the factors influencing the bearing capacity of the support. Zhao Feng et al.^11^ proposed a leverage effect model for the failure of the shield beam based on the working condition of the hydraulic support with high frame and low use, simulated and analyzed the model, and concluded that the shield beam is extremely vulnerable to failure under the leverage effect model. Wan Lirong et al.^12^ used coal rock direct impulse to load the tail beam to study the dynamic response of the tail beam after bearing the impact load, which provided a reference quantity for the dynamic control of the caving mechanism. Li Qiang et al.^13^ simulated and analyzed the column hydraulic system characteristics of the hydraulic support during the column lifting process, the column lowering process and the impact load. They also obtained the dynamic response curves of the pressure of each cylinder of the column, the pressure and flow of the safety valve under the column raising process, the column lowering process and the impact load. Zeng Qingliang et al.^14^ studied the impact response of the hinge of the support when the coal gangue particles impacted the hydraulic support in the process of top coal caving mining based on ABAQUS and explored the stress difference at the hinge of the support. Xie Yunyue et al.^15^ studied the distribution law of the specific pressure of the bottom plate of a hydraulic support under a deep well impact load. Hu Xiangxun et al.^16^ used numerical calculations to analyze the influencing factors of static stability and dynamic stability of the support and provided a scheme to increase the stability of the support. Liu Wei et al.^17^ proposed a method to detect the coal gangue interface by using the vibration characteristics of the coal gangue falling impact steel plate. Zhang Ningbo et al.^18^ proposed the method of measuring and identifying the mixed gangue at the coal caving opening in the process of top coal caving by using the natural ray of the coal gangue. Jiang Lei et al.^19^ proposed a coal gangue recognition method by using the vibration signal of the tail beam, and the recognition performance was better than that of the conventional network model. Shan Pengfei et al.^20^ the image recognition method based on the improved Faster R-CNN algorithm was used to discriminate the coal gangue release status, which provided theoretical support for the accurate recognition of coal caving.

The above literature has not involved research on the dynamic response of hydraulic support in the process of elastic coal and rock mass impact tail beam crushing. At the same time, there are few studies about the best installation position of information acquisition sensors in a fully mechanized top coal caving coal and rock identification, and the reliability of the data acquisition is low. According to the characteristics of top coal caving mining technology, this paper fully considers the elastic–plastic behavior of the coal-rock mass and the deformation and fracture behavior produced in the process of impacting the tail beam. Taking ZFY21000-35.5-70D top coal caving support as the engineering object, this paper analyzes the dynamic response of the support after a coal-rock impacts the tail beam and the best installation position of the coal caving monitoring sensor and adopts the means of combining theoretical analysis and numerical simulation. Based on the two-way coupling technology of EDM-FMBD, the virtual caving coal wall is established by using the discrete element software, EDEM, and the rigid flexible coupling model of the tail beam of the top coal caving support is established by using the multibody dynamics software, RecurDyn. The application of the solid–liquid spring coupling theory, equivalent column, balance jack, tail beam jack stiffness and spring replacement simulate the coal rock collapse impact tail beam process. It is found that the force of hinge point of tail beam shield beam and the force of hinge point above rear link change in the direction of complementary length, the hinge point above front link is the best installation position of force sensor to monitor the force during coal caving. Additionally, it can reduce the measurement error caused by the difference of the impact position. The hinge joint of the tail beam is selected as the best installation position of the angular acceleration sensor, which is combined with the tail beam abdominal vibration sensor to monitor the vibration signal in the process of coal caving. The research results have practical importance for coal gangue identification and support the adaptive control in fully mechanized top coal caving mining.

## Theoretical foundation

### Dynamic model of the coal rock impact tail beam

**Figure 1:**
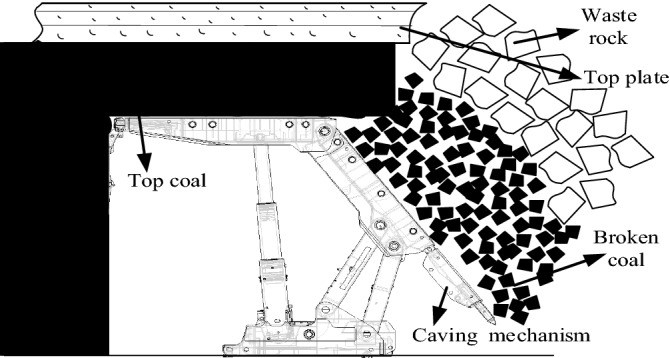
Mechanical model of the top coal caving support.

The overall mechanical model of the top coal caving support is shown in Fig. 1. It can be seen from the figure that in the process of top coal caving, the broken coal rock rolls and slides after impacting the tail beam, and the mechanical coupling between the two is very complex.

**Figure 2:**
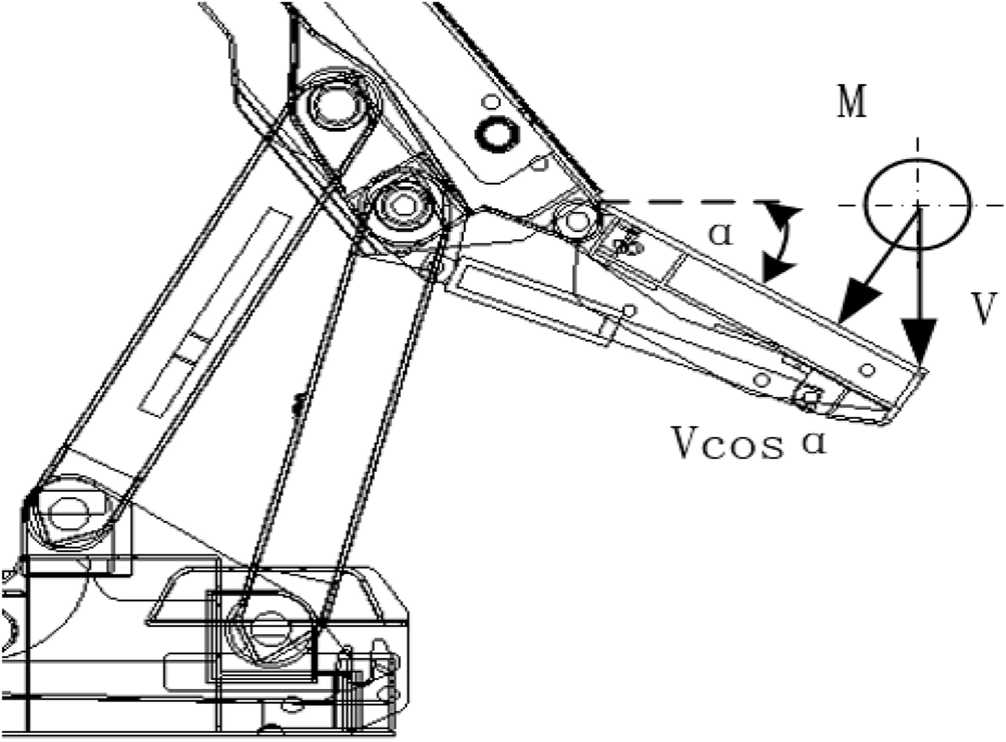
Simplified model of the coal rock impact tail beam.

To facilitate the research, the dynamic behavior of coal rock impacting the tail beam is equivalent to the coal rock particles with mass M colliding with the elastic metal plate at a certain impact speed V, and the simplified model is shown in Fig. 2. The energy of caving coal rock particles is partly converted into the elastic energy of particle collision and contact deformation and it is partly converted into the deformation energy of the metal plate. When the impact velocity of the coal rock particles is greater than its initial yield collision velocity, plastic strain will occur. The tail beam metal plate deformation energy, coal rock initial yield collision velocity, and maximum impact pressure can be obtained from Formulas (1)–(3):

1 $$S = \frac{F^{2}X^{3}}{3EI}$$

where *F* is the normal contact force, *X* is the position where the coal rock impacts the tail beam, and *EI* is the bending stiffness of the metal.

2 $$V_{q} = \sqrt {\frac{{32RE^{*2} X^{3} h^{3} }}{27MEI} + \frac{{16E^{*} R^{\frac{1}{2}} h^{\frac{5}{2}} }}{15M}}$$

3 $$J_{\max } = P + \pi RP\left( {h_{\max } - h_{q} } \right)$$

where *R* is the equivalent radius, E^*^ is the equivalent elastic modulus, *h* is the normal compression deformation, *h*_*q*_ is the normal yield and the pressing deformation, *h*_*max*_ is the maximum normal pressing deformation, *M* is the coal rock mass, and *P* is the initial yield stress.

### Coal rock contact model

The process of a coal rock collapse impacting the tail beam involves the contact between coal rock particles and particles and between particles and the tail beam. The contact and collision between particles leads to a change in energy and an impact response to the tail beam. In this paper, the Hertz-Mindlin model with bonding is selected as the contact model between the coal and rock particles, and its contact parameters can be obtained from Formulas (4)–(7):

4 $$K_{n} = \frac{2E}{{3\left( {1 - \mu^{2} } \right)}}\left( {R^{ * } } \right)^{\frac{1}{2}}$$

5 $$K_{S} = \left( {\frac{E}{1 + \mu }} \right)^{\frac{2}{3}} \frac{{\left[ {12\left( {1 - \mu } \right)R^{ * } F_{n} } \right]^{\frac{1}{3}} }}{2 - \mu }$$

6 $$F_{n} = K_{n} U_{n}^{\frac{3}{2}}$$

7 $$F_{S} = K_{S} U_{S}^{\frac{3}{2}}$$

where *K*_*n*_ is the normal stiffness, *K*_*S*_ is the tangential stiffness, *F*_*n*_ is the normal stress, *F*_*S*_ is the tangential stress, *E* is the elastic modulus of the particles, *μ* is Poisson's ratio, R^*^ is the particle contact radius, *U*_*n*_ is the particle normal displacement, and *U*_*S*_ is the particle tangential displacement.

### Solid–liquid spring coupling model

The column and the tail beam jack contain emulsions, which have a certain buffer effect. To facilitate the simulation analysis, the hydraulic system is equivalent to a solid–liquid spring coupling system, the emulsion in the hydraulic cylinder and the cylinder block are regarded as springs, and the equivalent stiffness calculation formula is in ^21^:

8 $$K = \frac{{K_{1} K_{2} }}{{K_{1} + K_{2} }}$$

where *K*_*1*_* K*_*2*_ is the equivalent stiffness of the emulsion and the stiffness of the cylinder block,

9 $$K_{1} = \frac{S}{{LK_{V} }}$$

10 $$K_{2} = \frac{{K_{gV} \pi d^{2} }}{4} = 2\delta E$$

where *S*, *L*, *K*_*V*_, _*kgV*_, *d*, *E*, and *δ* are the liquid column cross-sectional area, liquid column height, volume compression coefficient, cylinder elastic coefficient, cylinder inner diameter, cylinder elastic modulus and wall thickness, respectively. Formula (11) can be obtained from Formulas (8)–(10):

11 $$K = \frac{2S\delta E}{{S + 2LK_{V} \delta E}}$$

## Construction of the EDM-FMBD two-way coupling simulation model

**Figure 3:**
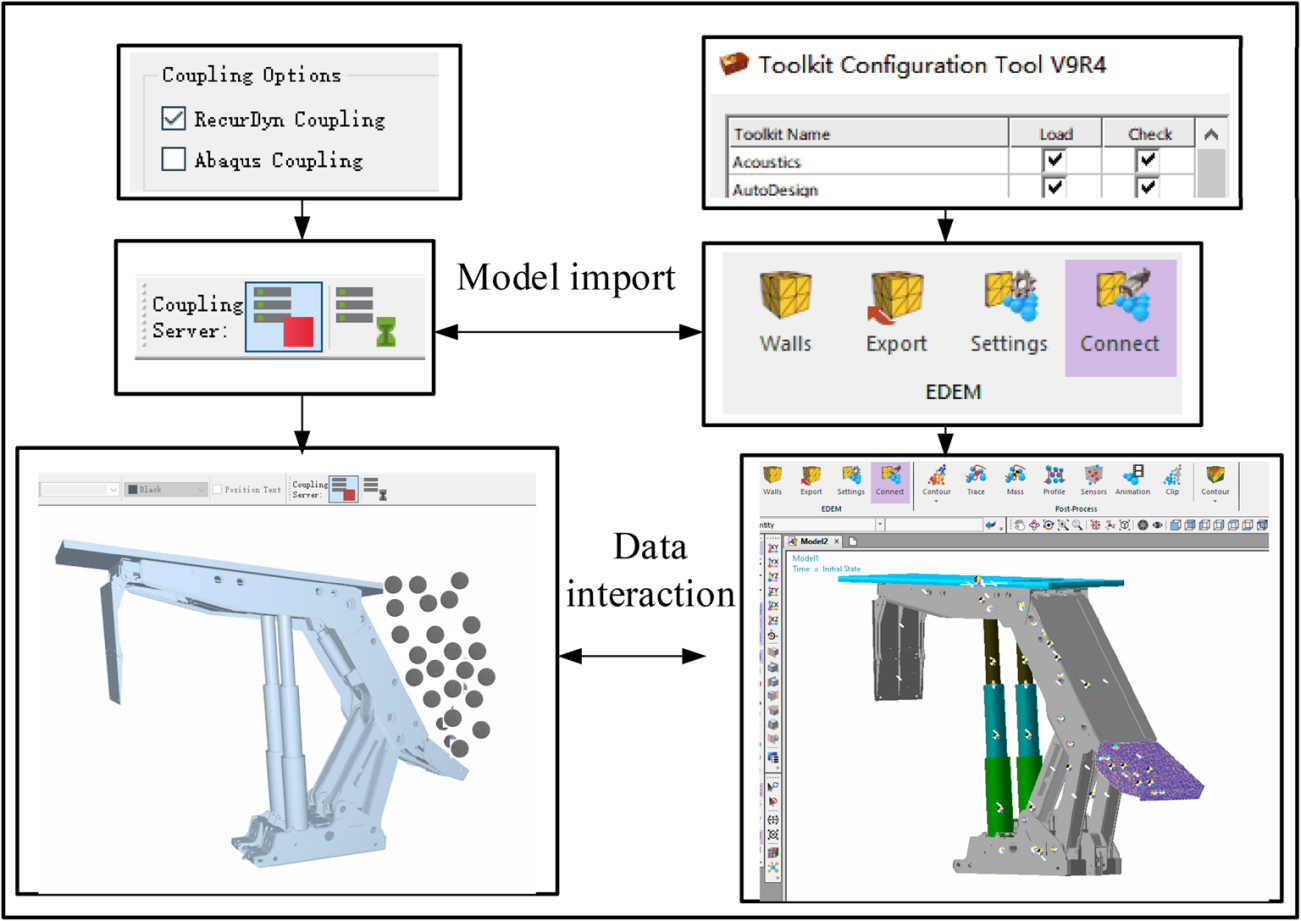
Bidirectional coupling process diagram.

As the coal caving mechanism of the top coal caving support, the tail beam produces violent collision and friction with coal rock in the process of coal caving and recovery, resulting in obvious vibration at the tail beam and each hinge; the mechanical transmission characteristics of the support will also change substantially. To accurately analyze the dynamic response signal after the coal rock impacts the tail beam, a DEM-MFBD two-way coupling system of discrete element bonding broken coal and top coal caving support is built, and its two-way coupling process is shown in Fig. 3.

### Construction of the discrete element model of the coal-rock

According to the occurrence conditions of the coal seams in the working face of the Yankuang Group, the physical and the mechanical properties of the coal and rock are measured for the samples taken according to the sampling standard, and the mechanical property parameters required for the experiment are shown in Table 1.

**Table 1:** Physical and mechanical parameters of the coal and rock.

Material	Density (kg·(m^3^)^−1^)	Elastic modulus (MPa)	Poisson ratio (μ)	Compressive strength (MPa)
Coal	1280	2010	0.28	12
Rock	2460	3260	0.24	30

**Figure 4:**
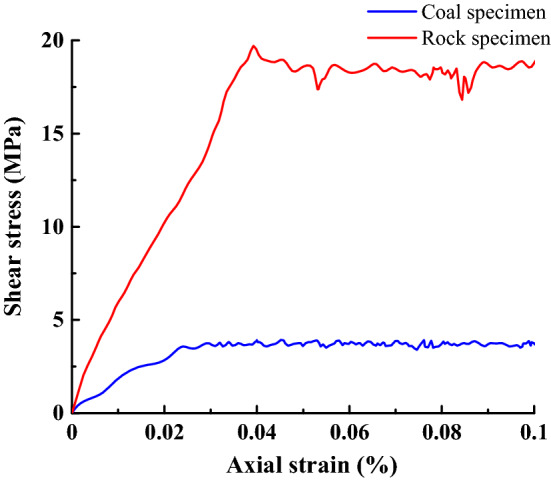
Stress–strain relationship.

Based on the actual working conditions, the EDEM software API method was used to construct the coal block model of multiparticle bonding. The radius of the impacted coal bonding particles is set to 12 mm^22^. Based on the measurement results of the coal rock physical and mechanical property parameters and the BP neural network, the bonding parameters between particles are obtained by uniaxial compression and the Brazilian splitting simulation test^23^, as shown in Table 2. The stress–strain curve of coal rock sample is shown in Fig. 4.

**Table 2:** Bonding parameters between particles.

Name	Normal stiffness (N·(m^3^)^−1^)	Tangential stiffness(N (m^3^)^−1^)	Normal maximum stress (Pa)	Tangential maximum stress (Pa)
Coal and coal	1.2165 × 10^8^	9.7320 × 10^7^	8.3183 × 10^6^	2.3573 × 10^6^
Coal and rock	1.5519 × 10^8^	1.2415 × 10^8^	1.7003 × 10^7^	7.5010 × 10^6^

### Construction of a rigid flexible coupling model of the tail beam of the top coal caving support

**Figure 5:**
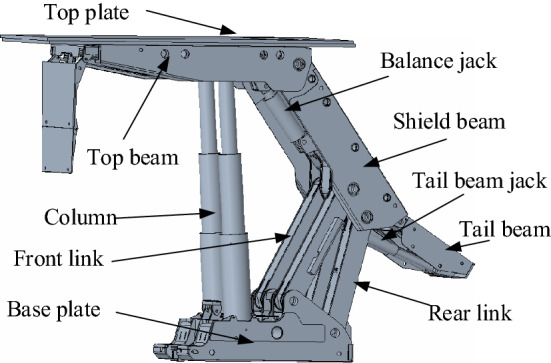
3D solid model of support.

The main parameters of the ZFY21000-35.5-70D top coal caving support are shown in Table 3. To ensure that underground mine test conditions are the same as the virtual prototype simulation, 1:1 3D modeling of the support is conducted. The 3D solid model was drawn by Creo8.0 software, as shown in Fig. 5.

**Table 3:** Main technical parameters of hydraulic support.

Support style		Two-leg sublevel caving shield support	
Support strength (MPa)		1.65–1.70	
Width (mm)	1.95–2.2	Center distance (mm)	2050
Initial support force (N)	1.65 × 10^7^	Eorking resistance(N)	2.1 × 10^7^
Floor specific pressure (MPa)	2.6–4.9	Height (m)	3.55/7.0
Pumping station pressure (MPa)	37.5	Weight (t)	78.9

**Figure 6:**
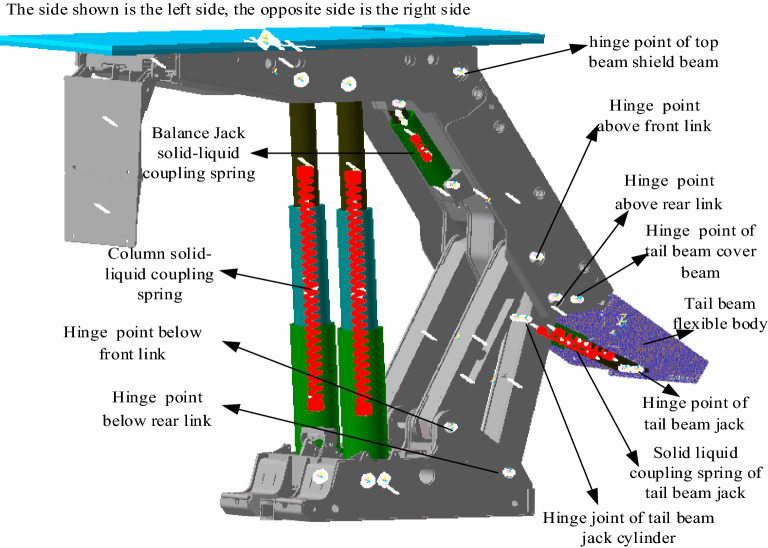
Rigid flexible coupling model of support.

The 3D solid model of the hydraulic support is imported into the RecurDyn software, and constraints are added based on the relative motion relationships between the main components. In the process of actual coal caving, the tail beam is directly involved in the impact collision and the roll down of the coal rock; then, it is very easy to wear and deform, thus changing the dynamic response characteristics of the support so that the tail beam will be flexible. The solid–liquid spring coupling method is used for equivalent replacement of the support column, balance jack and tail beam jack. According to Eq. (11), the equivalent stiffness coefficients of the balance jack, column and tail beam jack are 7.3 × 10^8^ N/m, 8.79 × 10^7^ N/m, and 1.02 × 10^8^ N/m, respectively. The main technical parameters of the ZFY21000-35.5-70D top coal caving support are shown in Table 3. From Table 3, the maximum working resistance of the support is 21,000 kN, and the roof pressure is 16,000 kN to simulate a stable working state of the support^21^. In the model, the preload of the column spring is equivalent to replacing the initial support force of the support. From the initial support force of the support, the preload of the column spring is 8272 kN. The rigid flexible coupling model of the tail beam of the coal rock impact crushing top coal caving support is finally established, as shown in Fig. 6.

### Solid–liquid spring coupling model and its validation

To verify the accuracy of the solid–liquid spring coupling model, a single coal ball impact tail beam mechanical-hydraulic co-simulation model was built.

**Figure 7:**
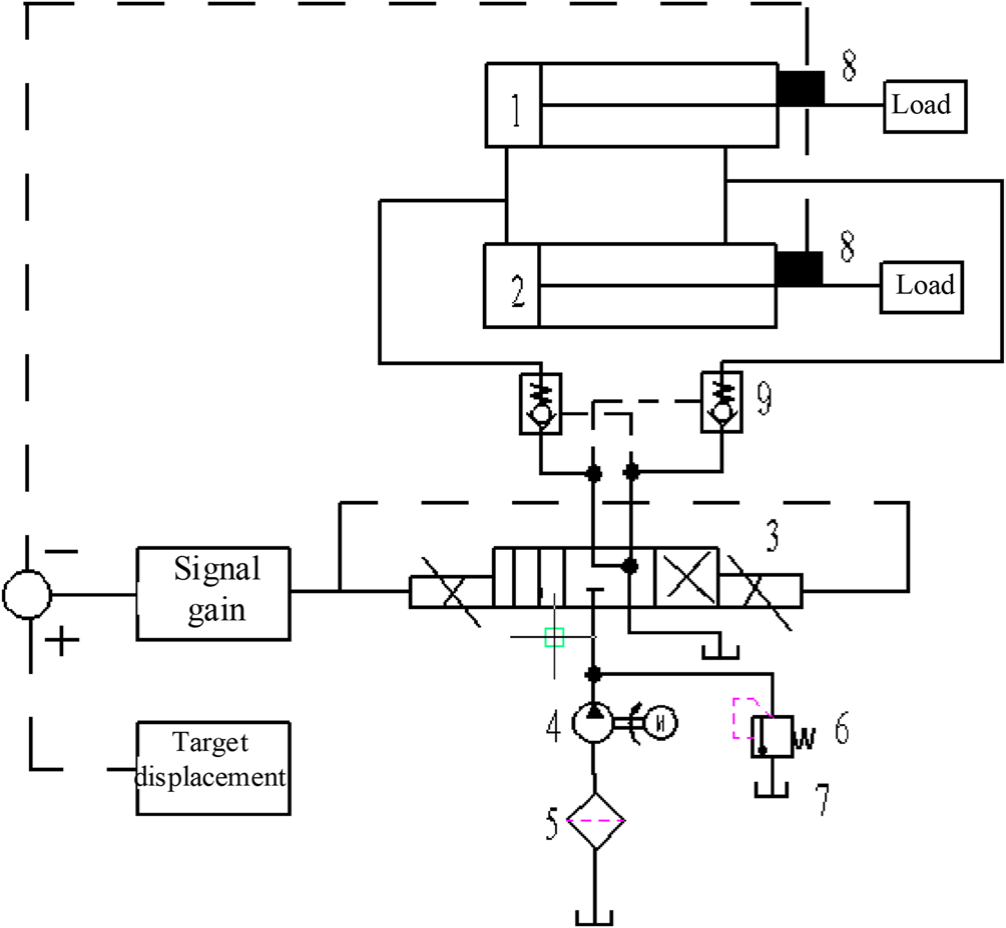
Structural diagram of the tail beam hydraulic control system. 1, 2—Tail beam jack; 3—Y-type electromagnetic proportional directional valve; 4—hydraulic pump; 5—filter; 6—relief valve; 7—oil tank; 8—displacement sensor; 9—hydraulic operated check valve.

The structure of the hydraulic control system of the tail beam of the top coal caving support is shown in Fig. 7. The dynamic medium of the hydraulic cylinder is an emulsion, and the bulk elastic modulus is 5 × 10^8^ pa. The main parameters of the hydraulic system are shown in Table 4.

**Table 4:** Main parameters of the hydraulic system.

Hydraulic cylinder		Cylinder diameter (mm)	Piston diameter (mm)	Oil chamber length (mm)
Column	First degree	530	500	1760
	Second grade	380	355	1590
Balance jack		320	230	200
Tail beam jack		230	140	546

**Figure 8:**
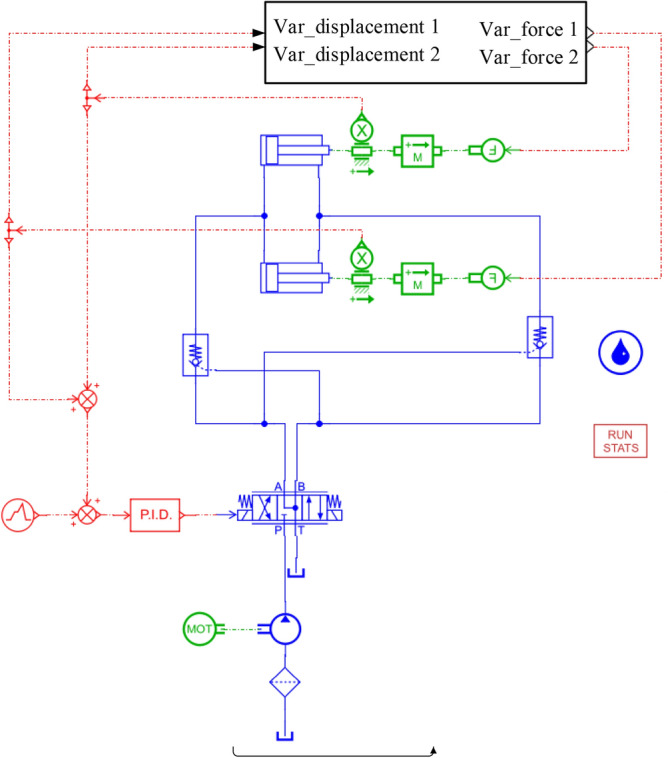
AMESim model of the hydraulic system.

According to the structure of the hydraulic system of the tail beam and the working principle of the coal caving of the coal caving support, a co-simulation model is established in AMESim as shown in Fig. 8, where the displacement of the tail beam jack piston is input from AMEsim to the kinematical pair of the RecurDyn hydraulic cylinder, and the RecurDyn inputs the tail beam jack force as output to the hydraulic cylinder piston in AMEsim. With the RecurDyn as the main control platform, after completing the interface arrangement, co-simulation begins. After the simulation, the maximum contact force and the cylinder force are selected for comparison with the maximum contact force and the spring force of the solid–liquid spring coupling simulation, and the errors are displayed in Table 5, which shows that the solid–liquid spring coupling error is less than 5%, indicating that it has a very high confidence level and meets the accuracy requirements.

**Table 5:** Error of solid–liquid spring coupling.

Name	Mechanical-hydraulic simulation	Solid–liquid coupling	Error (%)
Contact force (N)	9.3009 × 10^6^	8.8419 × 10^6^	4.93
Cylinder force (N)	1.1704 × 10^6^	1.1973 × 10^6^	2.699

## Analysis of numerical simulation results

### Dynamic response signal extraction

**Figure 9:**
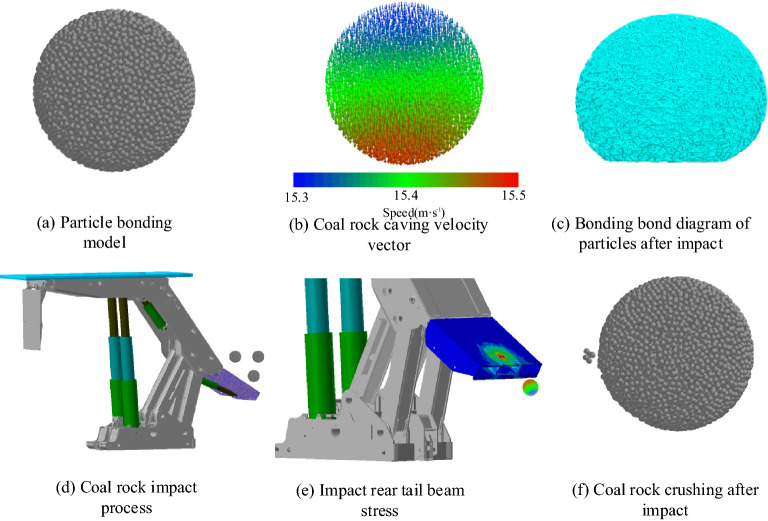
Numerical simulation process and results.

In the virtual prototype test of the coal rock impact tail beam, the coal rock body deforms elastically after the coal rock impacts the tail beam, cracks and breaks, and the stress at the impact point of the tail beam decreases in a circular shape one-by-one, accompanied by a violent vibration and the rolling friction of the tail beam. Combined with the support height and mining and caving ratio, a sphere with a radius of 200 mm is selected to impact the tail beam, with an impact height of 800 mm and a speed of 15 m/s. The simulation process and the results are shown in Fig. 9.

To accurately express the impact point location, a coordinate system, XOY, is established at the center of the upper surface of the tail beam, where the X and Y axes are parallel to the length direction and the width direction of the tail beam, respectively. After the two-way coupling simulation, the EDEM postprocessing module is used to view the coal rock fragmentation after impact, measure the coordinates at the moment of impact, and extract the force and acceleration amplitude of each articulation point in the RecurDyn postprocessing interface.

### Analysis of impact force transmission characteristics

To study the force variation trend of the hydraulic support under the action of impact loading, the data measured by the test are simplified, and the maximum peak value of the dynamic force of the hinge joint and the ratio of the force difference before impact to the force before the impact are defined as the force variation coefficient^24^ in formula (12):

12 $$\zeta_{{\left( {x,y} \right)}} = \frac{{F_{{2\max \left( {x,y} \right)}} - F_{1} }}{{F_{1} }}$$

where *ζ*_(*x,y*)_ is the force variation coefficient and (*x,y*) represents the impact position of the coal rock; *F*_*1*_ is the front force of the impact load; *F*_*2max*(*x,y*)_ is the maximum force after the impact load.

**Figure 10:**
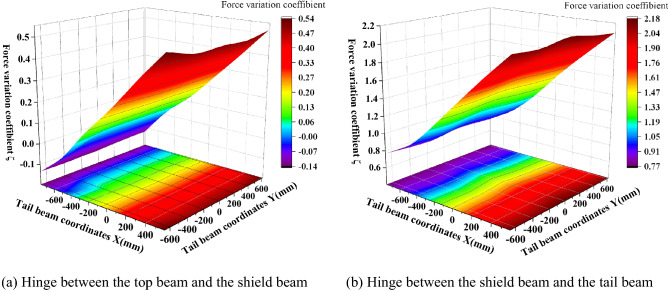
Force change of the hinge point between the beam and the shield beam, the shield beam and the tail beam.

When the coal rock caving impact acts on the tail beam at different positions, the force variation coefficients between the top beam of the support beam and the shield beam and between the shield beam and the tail beam hinge point are shown in Fig. 10. As seen in Fig. 10, the force variation coefficient is roughly symmetrical about the left and the right of the support and increases from the front end to the rear end of the tail beam along the direction of the length. This is because each hinge point is the moment of fulcrum, the impact load at the rear end of the tail beam has a long force arm, and the moment produced on the pivot point is also the largest. The force variation coefficient of the top beam and the shield beam hinge point is the smallest, and when the impact point is in front of the tail beam, a negative variation coefficient appears, that is, the force is decreasing. This occurs because when the impact point is near the tail beam hinge point, the tail beam swing amplitude is small and the distance between the top beam, the shield beam hinge point and the impact point is far and the impact energy loss is greater, which causes a negative variation coefficient. The force variation coefficient at the hinge point between the shield beam and the tail beam increases from 0.77 to 2.18 from the front end to the rear end of the tail beam; it increases slowly from the hinge point to the vicinity of X = − 450 mm and then rises rapidly.

**Figure 11:**
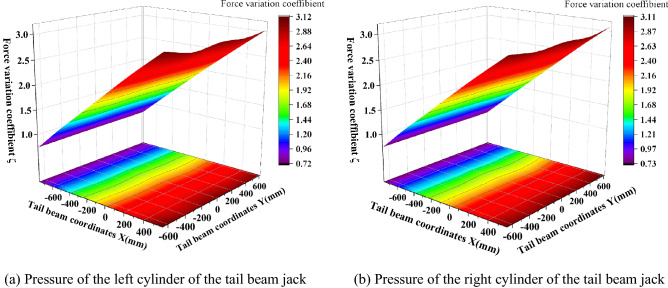
Tail beam jack pressure change.

The tail beam jack cylinder pressure changes, as shown in Fig. 11. Due to structural symmetry, the jack left cylinder pressure and the right cylinder pressure change coefficient remain basically the same from 0.7 to 3.1, and with a constant growth rate up.

**Figure 12:**
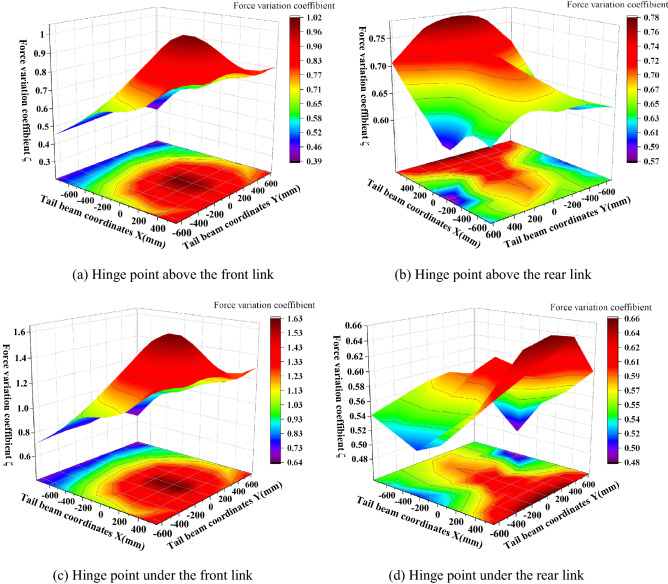
Force change of the front and rear link hinge points.

The coefficient of the variation of the front and rear link hinge point force is shown in Fig. 12. As seen in Fig. 12, the maximum coefficient of the variation of the front link upper and lower hinge point force appears near the center of the connecting line of the left and right piston rod hinge points of the tail beam jack with maximum values of 1.02 and 1.63, respectively, and gradually decreases outward from the center of the maximum value point in the shape of a mountain peak, with the smallest force variation coefficient near the tail beam hinge point. This transfer characteristic occurs because the impact point near the piston rod can form two force arms about the fulcrum of the front hinged point, which are from the tail beam to the shield beam and from the tail beam jack to the shield beam. The coefficient of the force variation at the hinge point on the rear link is more special, and the coefficient of the variation near the hinge point of the tail beam is the largest, gradually decreasing from the front end of the tail beam to the rear end, which is due to the closer distance between the hinge point on the rear link, the hinge point of the tail beam and the small loss of impact energy. The maximum and minimum values of the hinge point under the rear link are 0.66 and 0.48, respectively, and the coefficient of variation increases along the length direction. A small peak appears near the connecting line of the hinge joint of the left and right piston rods of the tail beam jack, and then the highest point at the rear end of the tail beam, which is caused by the interaction of the tail beam force arm and the tail beam jack force arm. In addition, the influence of the tail beam force arm is greater than that of the tail beam jack. The coefficient of the force variation of the four groups of hinge points along the width direction is more obvious, which is mainly because the width of the tail beam is 1.95 m, and the torque change caused by the width change is large.

**Figure 13:**
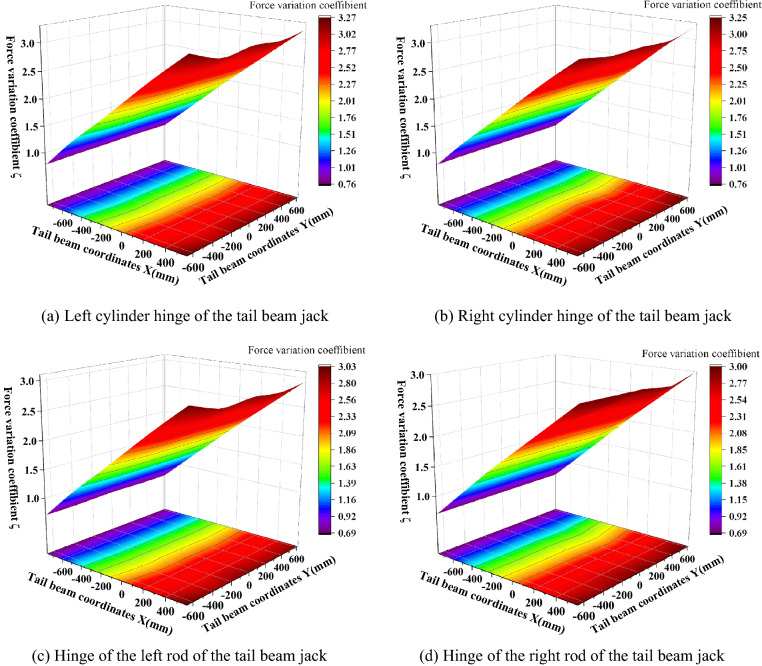
Force change of the tail beam jack hinge point.

The force variation coefficient of the tail beam jack cylinder and the piston rod hinge point is shown in Fig. 13. As seen in Fig. 13, the force variation coefficient at the hinge point of the tail beam jack increases gradually at a constant rate as the impact point moves toward the rear end of the tail beam. The force variation coefficient at the hinge point of the cylinder is greater than the force variation coefficient at the hinge point of the piston rod, and the maximum and minimum values are 3.2 and 0.7, respectively. This is because the force variation coefficient at the hinge point of the left and the right cylinders is basically the same due to structural symmetry.

### Analysis of the vibration characteristics of the hinge points

**Figure 14:**
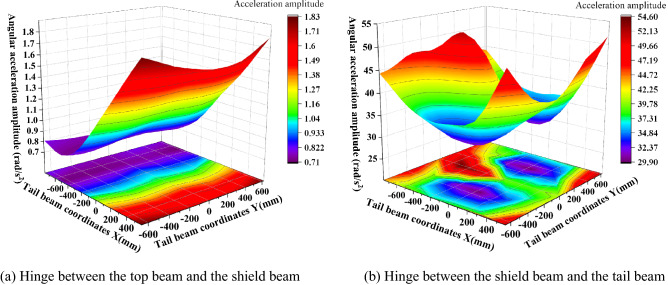
Amplitude of the hinge joint between the top beam and the shield beam, the shield beam and the tail beam.

The hinge joints of the hydraulic support are the key to connecting the components of the support and transferring the force. To study the change characteristics of the angular acceleration of each hinge point after the tail beam is impacted by different positions, the measured angular acceleration amplitudes of the top beam and the shield beam, the shield beam and the tail beam, and the front and rear link and the tail beam jack hinge points after the tail beam is impacted by the coal rock are processed and analyzed, as shown in Fig. 14. As seen from Fig. 14, the maximum and minimum values of amplitude at the hinge point of the top beam and the shield beam are 1.83 rad/s^2^ and 0.71 rad/s^2^, respectively, which appear near the hinge point of the tail beam and at the rear end of the tail beam, and the general change shows a trend that the amplitude gradually increases as the impact point moves toward the rear end of the tail beam, with the center of the width centerline gradually increasing to both sides. This change is caused by the increased torque at the hinge point between the top beam and the shield beam as the impact point moves toward the rear end of the tail beam. The overall amplitude of the hinge point of the tail beam and the shield beam is too great between 29.9 and 54.6 rad/s^2^. It is symmetrical with the centerline of the width and the change form of the "large at both ends and small in the middle" in the length direction, which is due to the front impact point being close to the hinge joint of the tail beam and the vibration being strong. However, as the impact point moves toward the rear end of the tail beam, the torque of the impact force on the hinge point of the tail beam increases and the angular acceleration amplitude is pulled up again.

The shield beam and the tail beam hinge point amplitude in the tail beam surface center of a region produce a very small value point with critical characteristics, so this region is called "amplitude low band." The impact point in the "amplitude low band" above, due to the impact point being close to the hinge point, the impact energy generated on the hinge point is strong and large in amplitude. When the impact point is below the "low amplitude zone," we take the moment from the hinge point as a fulcrum, and as the impact point moves to the rear end of the tail beam, the force arm increases and the torque on the fulcrum increases as the amplitude rises. When the impact point in the "low amplitude band" on the above two factors is less and this area is in the tail beam jack hinge point above, the cylinder will consume part of the collision energy.

**Figure 15:**
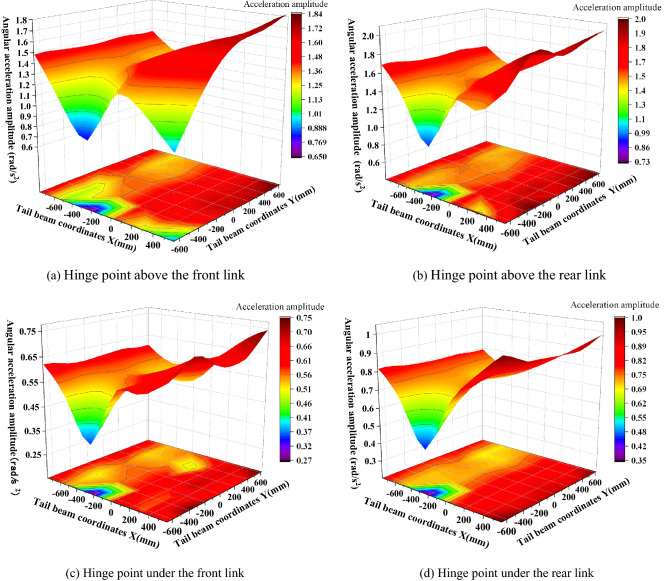
Amplitude change of the front and the rear link hinge points.

**Figure 16:**
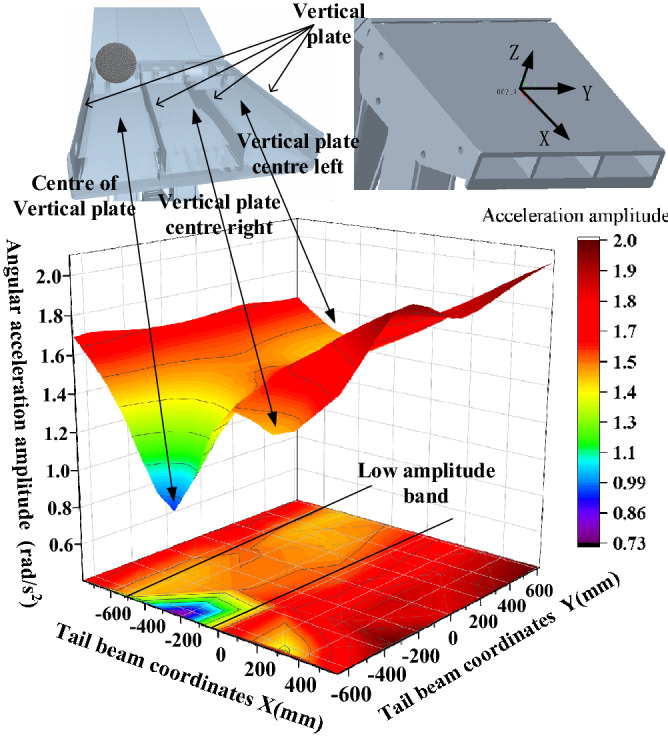
Analysis of the amplitude of the hinge point on the rear link.

The amplitudes of the front and rear hinge points are shown in Fig. 15. As seen in Fig. 15, the amplitude of the hinge point at the upper end of the connecting rod is greater than the amplitude of the hinge point at the lower end, and the amplitude of the rear link is greater than the amplitude of the front link, which is caused by the distance between the impact point and the hinge point. This is caused by the distance between the impact point and the hinge point, and the impact energy close to the impact point is strong and the amplitude is large. The amplitude of the four hinge positions is basically the same; the most obvious amplitude is the rear link hinge point, and the maximum and minimum values are 2.05 rad/s^2^ and 0.73 rad/s^2^, respectively. The length direction shows a "large at both ends and small in the middle" change pattern, and the impact torque effect at the rear end of the tail beam is greater than the impact effect at close range. For example, for the hinge point on the rear link, Fig. 16 shows that when the impact point is at the right end face of the tail beam, the amplitude has an obvious low point. By looking at the impact point coordinates and the three-dimensional model, the impact location is found to be just in the tail beam internal structure of the two vertical plate centers, where the elasticity of the upper plate of the tail beam is the largest. There is a certain buffering effect. In addition, the position is on the lower amplitude band in the front middle of the tail beam and produces the lowest point of the amplitude edge under the double action.

**Figure 17:**
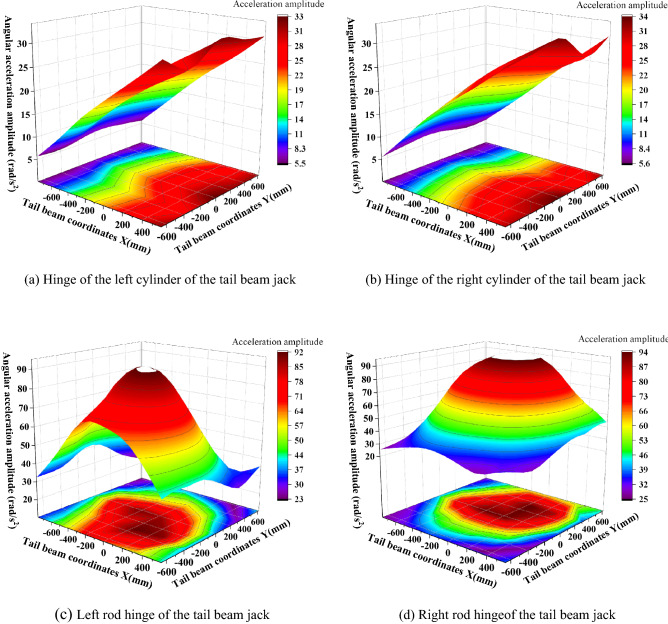
Amplitude change of the tail beam jack hinge point.

The amplitude of the hinge point of the tail beam jack is shown in Fig. 17. As seen in Fig. 17, the amplitude of the hinge point on the left and the right sides of the tail beam jack is basically the same due to the symmetry of the structure. Therefore, the left side is used as an example for analysis. The amplitude of the cylinder hinge point gradually increases as the impact point moves toward the rear end of the tail beam, and the amplitude ranges from 5.5 to 33.3 rad/s^2^. The piston rod hinge point amplitude changes from the highest point near the hinge point to gradually decrease outward in the shape of a mountain because the spring damping system near the connection of the region consumes more energy during the impact, and the hydraulic cylinder will absorb more energy, resulting in the piston rod hinge point amplitude becoming larger. The dynamic response volume achieves great values in these regions, forming an amplitude ring high region with amplitudes ranging from 22.8 to 92.0 rad/s^2^.

**Figure 18:**
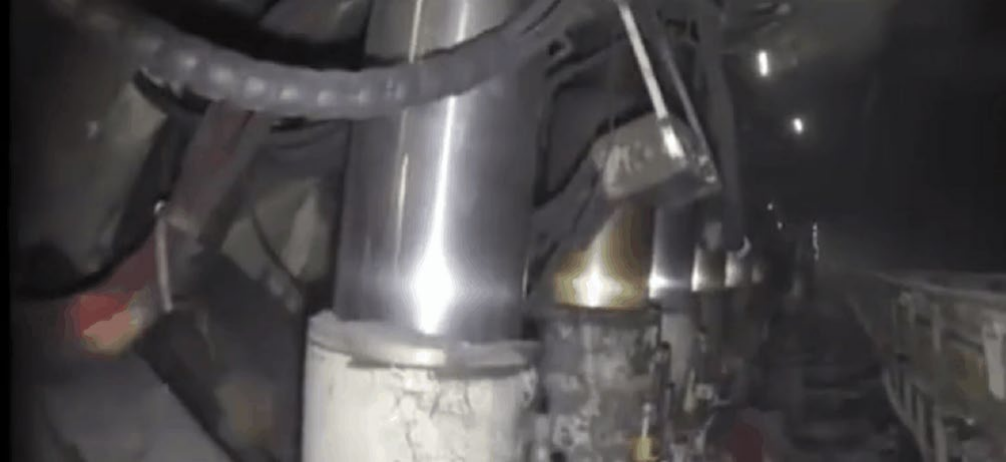
Underground experiment.

**Figure 19:**
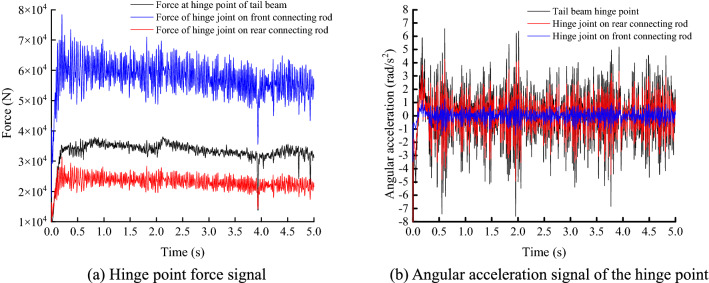
Collected data.

To verify the reliability of the virtual prototype test, the parameters of coal and rock of the 11302 working face of Yankuang Group Zhao Lou coal mine are calibrated, and the industrial tests were carried out. The 11302 working face of Yankuang Group Zhao Lou coal mine, using one cut one falling, the mining craft of parallel working of sectional mining and caving. The coal mining height of working face is 3 m, caving ratio is 1:1.2, 96 supports of ZFY7200 type are used in the working face. The center distance of hydraulic support is 1.5 m. The angular acceleration sensors and the force sensors are arranged at the tail beam and the hinge point of the front and rear link for monitoring, and the experimental site is shown in Fig. 18. The hinge point force signal obtained from the information collected by the sensor is shown in Fig. 19a, the hinge point angular acceleration signal is shown in Fig. 19b, and the statistical values of hinge point acceleration and force are shown in Table 6.

**Table 6:** Statistical value.

Response values	Tail beam hinge point	Hinge point on front link	Hinge point on rear link
Maximum force (N)	38,089	78,421	31,368
Minimum force (N)	10,054	29,233	11,693
Maximum acceleration (rad/s^2^)	6.58	1.27	4.28
Minimum acceleration (rad/s^2^)	−7.9	−1.55	−5.17

The Fig. 19a and Table 6 show that the force amplitude of the hinge point of the front link is the largest, which is 75.5% greater than that of the tail beam hinge point and 150.0% greater than that of the hinge point of the rear link. According to Table 6 in Fig. 19b, the amplitude of the tail beam hinge point is the largest, followed by the hinge point on the rear link. The amplitude of the tail beam hinge point is 53.2% greater than that of the hinge point on the rear link; the amplitude of the tail beam hinge point is 413.5% greater than that of the hinge point on the front link. The test results show that the force signal of the hinge point on the front link is the strongest in the coal caving process. The angular acceleration signal at the hinge point of the tail beam is the strongest, which is consistent with the numerical simulation test of the impact at different positions of the tail beam. According to Fig. 11b, Fig. 12a,b, the maximum force change coefficient in the impact simulation test is the hinge point of the tail beam, followed by the hinge point on the front link, and finally the hinge point on the rear link. There is a difference in the order of force amplitude intensity from the underground test, and the reason for this difference is that some sensitive positions were not impacted in the simulation test. It can be seen from Figs. 14b, 15a,b that the maximum acceleration amplitude in impact simulation test is the hinge point of the tail beam, followed by the hinge point of the rear link and finally the hinge point of the front link, which is completely consistent with the results obtained from the underground test. The comprehensive test results show that the signal of hinge point force on front link is the strongest during coal caving. The angular acceleration signal at the hinge point of the tail beam is the strongest.

## Conclusion

To determine the best measuring point of the sensor information collection and provide the best installation position for coal rock identification in fully mechanized caving sensors in the future, numerical simulation tests and underground tests of impacts at different positions of the tail beam were carried out, and the study found the following:

1. The force variation coefficients at the hinge point of the top beam and the shield beam, the hinge point of the tail beam, and the hinge point of the tail beam jack increase as the impact point moves from the front end of the tail beam to the rear end. The front link upper and lower hinge points in the tail beam and the tail beam jack connection near the region exhibit a maximum point, the rear link upper hinge point in the tail beam hinge point near the coefficient of change is the largest, and the force change coefficient of the rear link lower hinge point with the impact point to the rear end of the tail beam gradually increases.
2. Different articulation points have different amplitudes during coal-rock impact. The total amplitude of the articulation point of the tail beam is larger, symmetrical to the centerline of the width, and shows a change in pattern of "large at both ends and small at the middle" in the direction of the length.
3. The definition of a "low amplitude band" on the surface of the tail beam is proposed. When the impact point is located in this area, the amplitude of the hinge point has critical characteristics, the impact energy and the torque have little effect and it is very easy to generate minimum points.
4. The hinge point on the front link is the optimum installation position for the force sensor and the hinge point of the tail beam is the optimum installation position for the angular acceleration sensor. Monitoring dynamic signals in the process of coal caving can effectively improve the reliability of gangue identification for fully mechanized coal caving and adaptive support control.
